# Association between Decreased Klotho Blood Levels and Organic Growth Hormone Deficiency in Children with Growth Impairment

**DOI:** 10.1371/journal.pone.0107174

**Published:** 2014-09-08

**Authors:** Ido Wolf, Shiri Shahmoon, Michal Ben Ami, Yael Levy-Shraga, Kineret Mazor-Aronovitch, Orit Pinhas-Hamiel, Yonatan Yeshayahu, Rina Hemi, Hannah Kanety, Tami Rubinek, Dalit Modan-Moses

**Affiliations:** 1 Institute of Oncology, Tel Aviv Sourasky Medical Center, Tel Aviv, Israel; 2 Sackler Faculty of Medicine, Tel Aviv University, Tel Aviv, Israel; 3 Pediatric Endocrinology and Diabetes Unit, The Edmond and Lily Safra Children’s Hospital, Chaim Sheba Medical Center, Tel-Hashomer, Ramat-Gan, Israel; 4 Institute of Endocrinology, Chaim Sheba Medical Center, Tel-Hashomer, Ramat-Gan, Israel; Children’s National Medical Center, Washington, United States of America

## Abstract

**Objective:**

Klotho is an aging-modulating protein expressed mainly in the kidneys and choroid plexus, which can also be shed, released into the circulation and act as a hormone. Klotho deficient mice are smaller compared to their wild-type counterparts and their somatotropes show marked atrophy and reduced number of secretory granules. Recent data also indicated an association between klotho levels and growth hormone (GH) levels in acromegaly. We aimed to study the association between klotho levels and GH deficiency (GHD) in children with growth impairment.

**Design:**

Prospective study comprising 99 children and adolescents (aged 9.0±3.7 years, 49 male) undergoing GH stimulation tests for short stature (height-SDS = −2.1±0.6). Klotho serum levels were measured using an α-klotho ELISA kit.

**Results:**

Klotho levels were significantly lower (p<0.001) among children with organic GHD (n = 11, 727±273 pg/ml) compared to both GH sufficient participants (n = 59, 1497±754 pg/ml) and those with idiopathic GHD (n = 29, 1645±778 pg/ml). The difference between GHS children and children with idiopathic GHD was not significant. Klotho levels positively correlated with IGF-1- standard deviation scores (SDS) (R = 0.45, p<0.001), but were not associated with gender, pubertal status, age or anthropometric measurements.

**Conclusions:**

We have shown, for the first time, an association between low serum klotho levels and organic GHD. If validated by additional studies, serum klotho may serve as novel biomarker of organic GHD.

## Background

Klotho is a transmembrane protein which can be cleaved, shed and act as a circulating hormone [1]. Klotho-deficient mice (*kl/kl*) show a shortened life span and multiple disorders resembling human aging [1], while overexpression of klotho increases lifespan [2]. High klotho expression was noted in the distal convoluted tubules in the kidney and the choroid plexus, but klotho is also expressed in various endocrine-related tissues including testes, ovaries and the pituitary [1]. Various activities of klotho have been described to date. Klotho is an essential cofactor for the binding of fibroblast growth factor (FGF) 23 to its cognate receptor, thus serving as a major regulator of phosphate homeostasis [3], [4]. Klotho can also enhance the activity of the calcium channels TRPV5/6, and is a potent inhibitor of the activity of the insulin and insulin growth factor (IGF)-1 pathways [2], [5], [6].

Several lines of evidence indicate that klotho functions as a circulating hormone. Klotho can be isolated from body fluids, including blood, urine and cerebro-spinal fluid (CSF) [7], intra-peritoneal injection of klotho to mice affects glucose metabolism, and overexpression of klotho in a single organ can rescue the *kl/kl* mice [1], [2]. Recently, a reliable method for measuring klotho levels has been developed, which enabled the assessment of klotho blood levels in healthy subjects and in various diseases states [8]. Klotho serum levels are higher in children and decrease with aging [8], [9], [10]. Among elderly subjects, reduced klotho levels may be associated with increased mortality, increased rate of cardiovascular disease and disability in daily living activities [11], [12], [13].

An interaction between klotho, linear growth and growth hormone (GH) secretion and activity has been suggested. Klotho-deficient mice are smaller compared to their wild-type counterparts and their somatotropes show marked atrophy and reduced number of secretory granules [1]. Recently, increased klotho levels were observed in patients with GH-secreting pituitary adenomas, which rapidly returned to normal following removal of the adenomas [14], [15]. A putative role of klotho as a mediator of neonatal growth has also been suggested [16]. Finally, a strong association between klotho and IGF-1 levels was recently noted in a study comprising 159 healthy children [17].

We aimed to study the association between klotho serum levels, anthropometric measurements (growth and weight parameters), and GH deficiency (GHD) in children with short stature and growth impairment.

## Methods

### Patients

The Pediatric Endocrinology Unit at the Edmond and Lily Safra Children’s Hospital serves as a regional referral center for children and adolescents with growth retardation. Ninety-nine children and adolescents referred for GH stimulation test between June 2012 and May 2013 were included in this study. Demographic and clinical data (including age, gender, parents’ heights and previous anthropometric measurements) were collected and all patients underwent clinical evaluation. Routine blood tests included blood count, chemistry (including calcium and phosphate levels and liver and kidney function tests) and thyroid function tests.

The study has been approved by the Human Investigations Committee of the Sheba Medical Center, and informed consent was obtained from all parents.

### Anthropometric measurements

All patients had height and weight measurements performed on the day of the stimulation tests, and body mass index (BMI) was calculated based on the formula: weight (kg)/height (m)^2^. Height, weight, and BMI standard deviation scores (SDS) were calculated using age and gender-specific growth data (based on the Centers for Disease Control and Prevention’s Year 2000 Growth Charts) (www.cdc.gov/growthcharts). These data have been found adequate for assessing Israeli children [18]. Mid-parental height SDS (MPH-SDS), or genetic target heights, were calculated by averaging the heights of the parents, as documented in the medical chart, and adjusting by adding 6.5 cm for male patients and subtracting 6.5 cm for females. Target heights were expressed as SDS using the above reference.

### Pubertal Status

Clinical signs of puberty were documented to allow differentiation between prepubertal and pubertal children. Pubertal staging was performed according to the method of Tanner [19], [20]. Prepubertal state was defined as absence of pubic hair development and testis volume less than 3 ml for males, and absence of pubic hair and breast development for females. Pubertal males were defined as those with pubic hair development or testis volume more than 3 ml, and pubertal females were defined as those with pubic hair or breast development.

### Stimulation tests for GH secretion and definition of GHD

Because of the pulsatile nature of GH secretion, random GH measurements have no value and current guidelines advise the use of GH provocative stimulation tests after an overnight fast according to standardized protocols. Up to 20% of children with normal growth and stature may test “deficient”, if a single stimulation test is used. Therefore, if GH is low on one test, a second, confirming test is performed [21], [22]. In the current study, one of three pharmacologic stimuli were used at the referring physicians’ discretion: Glucagon (intramuscular injection of 0.1 mg/kg to a maximum of 1 mg), clonidine (150 micg/m^2^ to a maximum of 150 micg), and arginine (0.5 g/kg to a maximum of 30 g). Blood samples for GH levels were taken at 0, 30, 60, 90, and 120 minutes for all tests, and for glucagon also at 150 and 180 minutes. If the study showed a peak GH of less than 7.5 micg/L, a second and different test was conducted. GHD was defined as peak GH <7.5 µg/L on two different stimulation tests. Traditionally, a peak GH concentration <10 µg/L on two different tests has been used to diagnose GHD [21]. However, following the implementation of the standardization of the IMMULITE systems GH assay with the recombinant IS 98/574 (see below) [23] in Israel in 2010, GH cutoff values were changed to 7.5 µg/L, to adjust for the change in assay performance. Patients with additional pituitary hormone deficiencies received stable replacement therapy (thyroxine, n = 2; glucocorticoids, n = 1) as needed prior to the stimulation test.

### Measurement of klotho, GH and IGF-1 serum levels

Blood samples were drawn after overnight fasting, centrifuged for 15 minutes at 2700 rpm, separated and frozen at –70°C until use. Klotho levels in the serum were analyzed using an α-klotho ELISA kit (Immuno-Biological Laboratories Co, Japan). The kit has been validated and widely used for the measurement of klotho levels [8], [10], [24]. Measurements were conducted according to the manufacturer instructions. The intra- and interassay coefficients of variation ranged from 2.7 to 9.8%. GH was measured by a chemiluminescent immunometric method (Immulite 2000, Siemens Medical Solutions Diagnostics (Los Angeles, CA, USA), using as a standard the recombinant IS 98/574, which is calibrated in mass and units [19]. The analytical sensitivity of the assays was 2.6 nmol/L and 0.01 µg/L and the inter-assay CV ranged from 3.7 to 8.1% and from 4.2 to 6.6%, respectively. IGF-1 was measured by a chemiluminescent immunometric method (Immulite 2000, Siemens Medical Solutions Diagnostics (Los Angeles, CA, USA). The analytical sensitivity of the assays was 2.6 nmol/L and the inter-assay CV ranged from 3.7 to 8.1%. IGF-1 levels were transformed to natural logarithm (ln) in order to achieve normal distribution, and standard deviation scores (IGF-1-SDS) for each subject were calculated as explained elsewhere [25].

### Data analysis

The study variables were compared between the study groups using one way ANOVA for continuous variables and the Chi-square (χ2) test for categorical variables. Square root transformation of klotho levels was used in order to achieve normal distribution. No transformation was needed for any of the other study variables. Pearson correlation coefficient was used to determine the relation between continuous variables. Results were considered significant if the two-sided p-value was <0.05. Calculations were performed using SPSS 15.0, a statistical software package.

## Results

### Patients’ characteristics

Ninety-nine children and adolescents (male = 49) undergoing GH stimulation tests for the evaluation of short stature or growth retardation were prospectively evaluated. Their clinical and anthropometric characteristics are presented in Table 1. Mean age at the time of klotho measurement was 9.0 years (range 1.2–17.4) and mean height-SDS was –2.1±0.6. All patients had normal liver, kidney and thyroid function tests at the time of the study. Based on the results of GH stimulation tests, 59 patients were classified as GH sufficient and 40 patients were classified as having GHD. Of these 40 patients, 29 had idiopathic GHD and 11 patients had organic GHD. Causes of organic GHD were brain tumors with cranial irradiation (medulloblastoma, n = 4; brain metastasis with cranial irradiation, n = 1; optic glioma = 1); craniophryingioma with panhypopituitarism (n = 1); pituitary hypoplasia (n = 2); empty sella syndrome (n = 1); CNS bleeding (n = 1).

**Table 1:** Clinical and anthropometric characteristics according to GH status$.

	*GH Sufficient*	*Idiopathic GHD*	*Organic GHD*	*p-value*
	(n = 59)	(n = 29)	(n = 11)	
**Age**	8.9±3.3	8.9±4.3	9.7±4.0	NS
**M/F**	29/30	15/14	5/6	NS
**Height-SDS**	–2.1±0.5	–1.9±0.7	–2.6±1.1	0.02*
**Weight-SDS**	–2.0±1.0	–1.2±1.3	–2.1±1.6	0.01**
**BMI-SDS**	–0.9±1.2	0.7±1.3	–0.2±1.2	0.009***
**MPH-SDS**	–0.8±0.8	–0.7±0.8	–0.4±1.1	NS
**Δ height-SDS**	–1.3±0.8	–1.2±1.1	–2.5±1.4	0.003*
**Pre-pubertal**	77.1%	71.4%	70.0%	NS
**bone age/chronological age**	0.79±0.2	0.76±0.16	0.83±0.19	NS

GH: growth hormone; GHD: growth hormone deficiency; Δ height-SDS: difference between participants’ height SDS and the genetic target height (height-SDS - MPH^‡^-SDS); BA/CA: ratio between bone age and chronological age.

$ Continuous variables were compared using one way ANOVA; categorical variables (gender, pubertal state) were compared using the Chi-square (χ2) test.

*Organic GHD lower than both Idiopathic GHD and GHS.

**Idiopathic GHD higher than both organic GHD and GHS.

***GHS significantly lower than IGHD.

‡ MPH: Mid-parental height (average of the heights of the parents, plus 6.5 cm for male patients and minus 6.5 cm for females).

No significant differences between the three groups were noted regarding age, gender, midparental height-SDS (MPH-SDS), bone age/chronological ratio (BA/CA) or Tanner staging (Table 1). However, children with organic GHD were significantly shorter compared to GH sufficient children or children with idiopathic GHD and were also significantly shorter compared to their parents (Table 1). Children with organic GHD also had lower IGF-1-SDS and Pi levels but higher calcium levels (Table 2).

**Table 2:** Laboratory measurements according to GH status.

	*GH Sufficient*	*IGHD*	*OGHD*	*p-value*
	(n = 59)	*(n = 29)*	*(n = 11)*	
**Glucose (mg/dl)**	74±8	75±14	72±9	NS
**Calcium (mg/dl)**	10.0±0.3	9.9±0.5	10.5±0.5	0.02**
**PO4 (mg/dl)**	5.0±0.6	4.9±0.6	4.1±0.3	0.03**
**Peak GH (µg/L)**	12.1±4.8	5.8±1.6	3.5±2.6	<0.001*
**IGF-1 SDS**	–1.4±1.5	–2.0±2.8	–3.7±2.5	0.004**
**Klotho (pg/ml)**	1645±778	1497±754	727±273	<0.001**

OGHD: organic growth hormone deficiency; IGHD: idiopathic growth hormone deficiency.

*GH sufficient compared to organic and idiopathic GHD.

**Organic GHD compared to both other groups.

### Klotho serum levels among study participants

**Figure 1:**
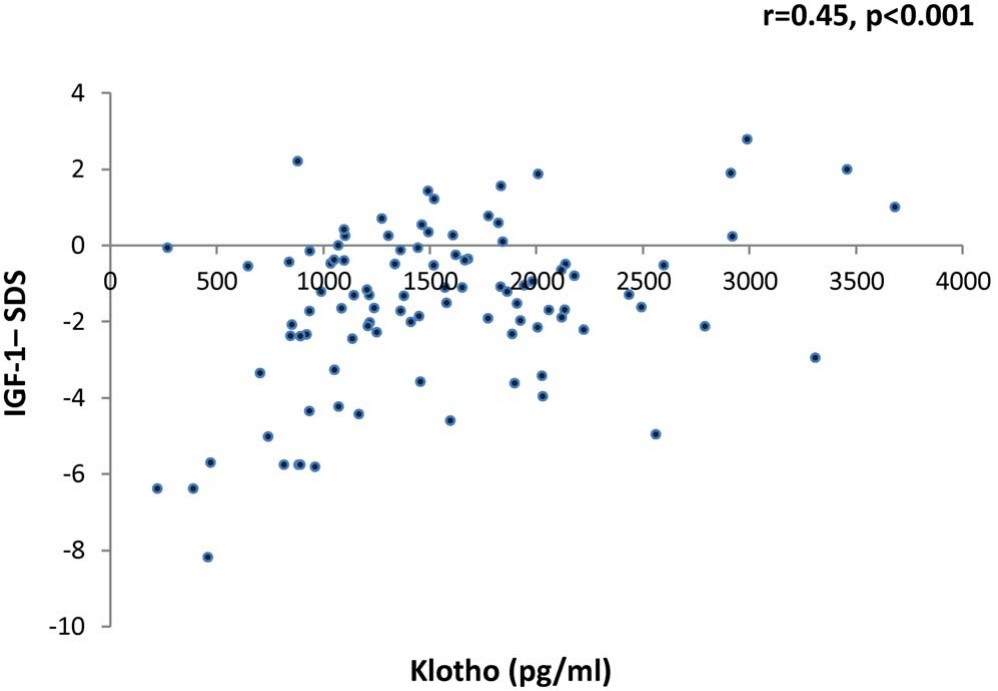
Correlation between serum klotho levels and IGF-1 (as assessed with the Pearson correlation coefficient).

Mean klotho level for the whole study group was 1500±754 pg/ml (range 220–5410). Klotho levels did not correlate with gender, age, pubertal status, or with anthropometric measurements, including height-SDS, weight-SDS and BMI-SDS (data not shown). However, klotho levels were significantly (p<0.001) lower among children with organic GHD (727±273 pg/ml) compared to both GH sufficient participants (1645±778 pg/ml) and those with idiopathic GHD (1497±754 pg/ml) (Table 2). The differences between GH sufficient children and children with idiopathic GHD were not statistically significant. Klotho levels were positively and significantly correlated with IGF-1 levels amongst all participants (r = 0.45, p<0.001, Figure 1). This correlation remained significant when patients with organic GHD were removed form the analysis (r = 0.27, p = 0.01).

Klotho serum levels did not correlate with serum phosphate levels, however a significant correlation between klotho and calcium levels was noted in GH sufficient children (r = 0.48, p = 0.03).

## Discussion

We assessed here, for the first time, the correlation between serum klotho levels, anthropometric measurements and GHD in a cohort of children undergoing GH stimulation testing for the evaluation of short stature and growth retardation. Our findings suggest a close association between klotho and the GH/IGF-I axis. Klotho levels in children with organic GHD were significantly lower compared with GH sufficient children and those with idiopathic GHD. Furthermore, klotho levels significantly correlated with IGF-1-SDS. Finally, we found no association between klotho levels and any of the anthropometric measurements, suggesting that low klotho levels were specifically associated with GH/IGF-I activity and not with short stature *per se*.

While our findings strengthen previous findings regarding the tight interaction between klotho and the GH/IGF-I axis, the nature of this interaction has not been yet elucidated, and current knowledge suggests that it may be bi-directional. In healthy children, IGF-1 levels were positively correlated with klotho levels [17]. Two recent studies in patients with acromegaly demonstrated increased blood levels of klotho which returned to normal after normalization of GH and IGF-1 levels [14], [15], suggesting that klotho expression or secretion is regulated by GH or IGF-I. Accordingly, insulin has been shown to mediate the cleavage and shedding of klotho from cell membranes into the circulation [26], and preliminary findings from our lab show a similar effect of IGF-1 on klotho cleavage (unpublished data). On the other hand, klotho may be a regulator of the GH/IGF-I axis. Klotho-deficient mice are smaller compared to their wild-type counterparts and their somatotropes show marked atrophy and reduced number of secretory granules [1]. In addition, klotho is a potent inhibitor of the IGF-I receptor [2], [5], [6], and IGF-I is a negative regulator of GH secretion. Therefore, reduced klotho levels may result in increased IGF-I signaling, causing reduced GH secretion. Taken together, current data suggest a putative negative feedback mechanism: increased IGF-1 levels enhance shedding of klotho, which in turn inhibits IGF-1 signaling.

Klotho levels of children with idiopathic GHD were somewhat lower compared to those of GH sufficient children, but the difference did not reach statistical significance. A possible explanation is the heterogeneity of the idiopathic GHD group. The diagnosis of GHD in children has been the subject of much debate. Currently, there is no “gold-standard” for the biochemical diagnosis of GHD, and the diagnosis requires comprehensive clinical assessment including height and weight measurements and evaluation of the growth rate, combined with biochemical tests of the GH-IGF axis [22]. As GH is secreted at intervals, random GH measurements are useless and the secretion is assessed using stimulation tests. There is a considerable overlap in peak GH concentrations between children with normal growth and those with GHD [18] and up to 20% of children with normal growth and stature may test “deficient” if a single stimulation test is used [27]. Furthermore, only 15–36% of patients with idiopathic GHD retest as GH deficient after discontinuation of GH treatment [28], [29], [30], [31]. It is therefore plausible that the idiopathic GHD group included children who were not truly GH deficient. Accordingly, both GH sufficient and idiopathic GHD groups had similar clinical and laboratory parameters and significantly differed only in their peak GH (Table 1, Table 2). Specifically, and in accordance with the similarity of klotho levels of the two groups, IGF-I and phosphate levels were also similar (Table 2).

Recent studies suggested a correlation between klotho and calcium levels among elderly patients but not in neonates, healthy children or patients with chronic kidney disease [8], [13], [16], [32]. However, no correlation between klotho and calcium serum levels was noted in a group of 181 healthy individuals of various ages [8]. We observed a slight elevation in calcium levels in the organic GHD group. Elevated calcium levels were noted in *kl/kl* mice and re-expression of klotho in these mice restored calcium to nearly normal levels [1]. Several mechanisms linking klotho to calcium levels have been suggested. More studies are needed in order to decipher the association between klotho and calcium blood levels.

We noted lower phosphate levels among participants with organic GDH compared to GH sufficient participants and those with IGHD. Low phosphate levels have previously been reported in children with GH deficiency, increasing following treatment with GH [33]. Correspondingly, increased phosphate levels were noted in acromegaly patients, returning to normal following successful surgery [34]. The detailed mechanism of these changes in phosphate levels remains to be elucidated, but the effect has been postulated to be klotho mediated. Ample data indicate a complex interaction between klotho, IGF-1 and FGF23. In healthy children, IGF-1 levels correlated with intact FGF23 levels [35], and in children with GHD C-terminal FGF23 levels (including both the active and the inactive form of FGF23) increase during GH treatment [36]. Consistently, increased FGF23 levels were also noted in some acromegaly patients [34]. However, no correlation between intact (active) FGF23 and phosphate levels were noted in acromegaly [34], healthy children [35] or children with chronic kidney disease [37]. A recent study investigated klotho, IGF-1 and FGF23 levels in 159 healthy children and noted no association between IGF-1 and FGF23 levels, and multivariate analysis indicated association between IGF-1, but not FGF23, with klotho levels [17]. Further research is needed in order to decipher the complex interactions between these factors in children with organic GHD.

Several limitations of this study should be acknowledged. Firstly, the study did not include a control group of normal-height children. An obligatory inclusion criterion for this study was the conduction of GH stimulation tests. Due to ethical limitations, these tests could not be done in normal children. However, klotho levels in the GH sufficient participants in our study were similar to those observed in healthy children [17]. Moreover, we noted no association between klotho levels and height-SDS. Thus, the lack of a control group of normal-height children does not affect the main conclusion of the study regarding the association between the GH/IGF-1 axis and klotho among children with short stature. Secondly, the number of patients in the organic GHD group was small and included children with several diagnoses. It is possible that the underlying disease also affected klotho levels. This should be tested in larger cohorts of patients with organic GHD. Finally, a wide array of mechanisms may mediate short stature. Thus, the GHS group likely comprised children with a heterogeneous group of etiologies. Still, due to the low false negative rates of the GH stimulation tests, it is likely that those who tested negative are indeed GHS [21], [22], [28].

In conclusion, we have shown, for the first time, an association between low soluble klotho levels and organic GHD. Whether low klotho levels are the cause or the result of GHD remains to be elucidated. If validated by additional studies, serum klotho may serve as novel biomarker of organic GHD.
